# Efficacy of travoprost for the treatment of patients with glaucoma

**DOI:** 10.1097/MD.0000000000016526

**Published:** 2019-07-19

**Authors:** Xiu-Li Zhang, Li Qin

**Affiliations:** Department of Ophthalmology, The First Affiliated Hospital of Xi’an Jiaotong University, Xi’an, China.

**Keywords:** efficacy, glaucoma, randomized controlled trial, safety, travoprost

## Abstract

**Background:**

This study will evaluate the efficacy of travoprost for patients with glaucoma systematically.

**Methods:**

A comprehensive literature search will be carried from following literature sources from inception to the present: Cochrane Library, MEDLINE, EMBASE, Web of Science, Google scholar, Chinese Biomedical Literature Database, and China National Knowledge Infrastructure. We will only consider randomized controlled trials on assessing the efficacy and safety of travoprost for glaucoma for inclusion. We will use Cochrane risk of bias tool for the methodological quality assessment for each qualified study. If it is possible, we will pool the outcome data, and will perform meta-analysis.

**Results:**

This study will systematically evaluate the efficacy and safety of travoprost for glaucoma. Primary outcomes include intraocular pressure (IOP), mean IOP, and mean reduction of IOP. Secondary outcomes consist of diastolic ocular perfusion pressure, central corneal thickness, and quality of life, as measured by 36-Item Short Form Health Survey, and treatment-related adverse events included hyperemia, eye pain, and eye pruritus.

**Conclusion:**

The findings of the present study will summarize the updated evidence of travoprost for patients with glaucoma.

**PROSPERO registration number:** PROSPERO CRD42019126956.

## 1 Introduction

Glaucoma is a chronic optic neuropathy, which is depicted by the alteration of the optic nerve and the death of retinal ganglion cells .^[1–3]^ It is one of the most leading causes of permanent blindness around the world ,^[4–6]^ and occurs most often in older adults .^[7–9]^ It consists of primary and secondary open-angle and angle-closure glaucoma, respectively.^[9]^ It has been estimated to affect 76million in 2020 and 112 million people in 2040.^[10]^

A variety of managements are utilized to treat glaucoma, including timolol, valproic acid, latanoprost, travoprost, and so on, especially for travoprost.^[11–18]^ Lots of previous studies have reported that travoprost can effectively treat glaucoma.^[19–25]^ However, no study has systematically explored its efficacy and safety for patients with glaucoma. Therefore, this study will systematically assess the efficacy and safety of travoprost for the treatment of patients with glaucoma.

## 2 Methods

### 2.1 Eligibility criteria for study selection

#### 2.1.1 Types of studies

This proposed study will include randomized controlled trials (RCTs) that have assessed all forms travoprost for patients with glaucoma. However, any other studies will be excluded, such as nonclinical trials, case studies, noncontrolled trials, non-RCTs, and quasi-RCTs.

#### 2.1.2 Types of participants

All participants with clinically diagnosed as glaucoma will be considered for inclusion in this study without restrictions of country, race, sex, age, educational background and economy status.

#### 2.1.3 Types of interventions

In experimental group, patients can receive any form of travoprost alone for patients with glaucoma. In the control group, patients can undergo any treatments, except travoprost.

#### 2.1.4 Types of outcomes

Primary outcomes include intraocular pressure (IOP), mean IOP, and mean reduction of IOP. Secondary outcomes include diastolic ocular perfusion pressure, central corneal thickness, and quality of life, as measured by 36-Item Short Form Health Survey, and treatment-related adverse events included hyperemia, eye pain, and eye pruritus.

### 2.2 Strategy of literature searches

We will conduct a comprehensive literature search from following literature sources from inception to the present: MEDLINE, EMBASE, Cochrane Library, Web of Science, Google scholar, Chinese Biomedical Literature Database, and China National Knowledge Infrastructure without any language restrictions. Any RCTs on assessing the efficacy and safety of travoprost for glaucoma will be fully considered for inclusion. Additionally, we will also manually search the dissertations, conference proceedings, and reference lists of included studies, and relevant reviews. The search strategy of Cochrane Library is shown in table 1 table 1. The identical search strategies for other electronic databases will also be built and applied.

**Table 1:**
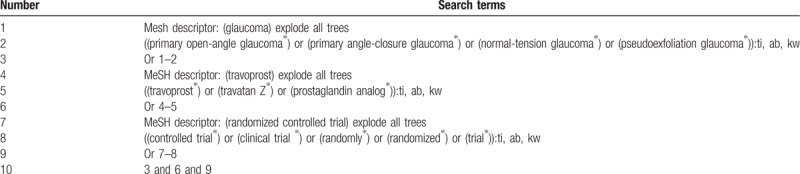
Search strategy applied in CENTRAL database.

### 2.3 Study selection

After removed duplication studies, 2 researchers will independently identify literature records through titles and abstracts initially. Then, the remaining studies will be further identified by reading full texts according to the predefined eligibility criteria. Any discrepancies will be solved with a third researcher through discussion. The process of study selection will be presented in a flow diagram.

### 2.4 Data extraction

All relevant data from eligible RCTs will be extracted and will be entered into a predesigned standardized data collection form by 2 researchers independently. Any discrepancies regarding the data collection will be resolved by a third researcher through discussion.

The standardized data collection form comprises of following information: General information, that is, first author name, publication year, title, country, among others; participant information, that is, diagnostic criteria, inclusion and exclusion criteria, age, sex, number of patients, and so on; study methods, that is, details of randomization, concealment, blinding, and so on; intervention details, that is dosage, frequency, duration, and so on; outcome details, that is, primary, secondary, and safety outcome measurements, among others.

### 2.5 Dealing with missing data

We will attempt to contact primary authors to obtain any insufficient or missing information from original eligible RCTs. If we cannot obtain those data, we will only analyze the available data. Meanwhile, we will also discuss its potential impacts.

### 2.6 Methodological quality assessment for eligible RCTs

Two researchers will independently assess methodological quality for all eligible RCTs by using Cochrane Collaboration Tool. It comprises of 7 domains, and each aspect will be graded as 3 types: low risk of bias, unclear risk of bias, and high risk of bias, respectively. Any disagreements will be settled down by a third researcher through discussion.

## 3 Statistical analysis

We will utilize RevMan 5.3 software to analyze the data. Binary valuables will be represented with risk ratio with 95% confidence intervals (CIs). Continuity changes will be represented with mean difference or standardized mean difference with 95% CIs. Heterogeneity among eligible trials will be identified using *I*^*2*^ test. When *I*^*2*^ ≤50%, heterogeneity is acceptable and a fixed-effect model will be applied. Data will be pooled and meta-analysis will be conducted if it is possible. When *I*^*2*^ >50%, heterogeneity is significant, and a random-effect model will be used. We will also perform subgroup analysis to detect any possible reasons that may contribute to the high heterogeneity based on the different treatments, controls, and outcomes. When the heterogeneity is still substantial after subgroup analysis, data will not be pooled, and meta-analysis will not be operated. Meanwhile, a narrative summary will be elaborated.

Additionally, sensitivity analysis will be carried out to investigate the robustness of pooled results by taking away low-quality trials. Whenever possible, we will also perform funnel plot^[26]^ and Egger regression^[27]^ to check any possible reporting bias if >10 eligible trials are entered in this study.

## 4 Discussion

Glaucoma is one of the most leading causes of permanent blindness. Travoprost is reported to treat glaucoma effectively. However, no study has systematically investigated the efficacy and safety of travoprost for the treatment of glaucoma. Thus, this study will assess the efficacy and safety of travoprost for glaucoma systematically.

This study will summarize a better understanding of efficacy and safety of travoprost for patients with glaucoma. The results of this study will inform our understanding of the value of travoprost in treating glaucoma outcomes. In addition, they will also provide helpful evidence for clinical practice and future researches.

## Author contributions

**Conceptualization:** Li Qin, Xiu-Li Zhang.

**Data curation:** Li Qin, Xiu-Li Zhang.

**Formal analysis:** Li Qin.

**Funding acquisition:** Xiu-Li Zhang.

**Investigation:** Li Qin.

**Methodology:** Li Qin, Xiu-Li Zhang.

**Project administration:** Li Qin.

**Resources:** Li Qin, Xiu-Li Zhang.

**Software:** Xiu-Li Zhang.

**Supervision:** Li Qin.

**Validation:** Li Qin, Xiu-Li Zhang.

**Visualization:** Li Qin, Xiu-Li Zhang.

**Writing – original draft:** Li Qin, Xiu-Li Zhang.

**Writing – review & editing:** Li Qin, Xiu-Li Zhang.
